# Bone indicators of grasping hands in lizards

**DOI:** 10.7717/peerj.1978

**Published:** 2016-05-02

**Authors:** Gabriela Fontanarrosa, Virginia Abdala

**Affiliations:** 1 Instituto de Biodiversidad Neotropical, UNT- CONICET, Tucuman, Argentina; 2 Facultad de Ciencias Naturales e IML, UNT, Cátedra de Biología General, Tucuman, Argentina

**Keywords:** Lizard, Hand anatomy, Grasping hand, Lizard hand skeleton, Prehensility, Narrow branches, Lizard hand morphometry

## Abstract

Grasping is one of a few adaptive mechanisms that, in conjunction with clinging, hooking, arm swinging, adhering, and flying, allowed for incursion into the arboreal eco-space. Little research has been done that addresses grasping as an enhanced manual ability in non-mammalian tetrapods, with the exception of studies comparing the anatomy of muscle and tendon structure. Previous studies showed that grasping abilities allow exploitation for narrow branch habitats and that this adaptation has clear osteological consequences. The objective of this work is to ascertain the existence of morphometric descriptors in the hand skeleton of lizards related to grasping functionality. A morphological matrix was constructed using 51 morphometric variables in 278 specimens, from 24 genera and 13 families of Squamata. To reduce the dimensions of the dataset and to organize the original variables into a simpler system, three PCAs (Principal Component Analyses) were performed using the subsets of (1) carpal variables, (2) metacarpal variables, and (3) phalanges variables. The variables that demonstrated the most significant contributions to the construction of the PCA synthetic variables were then used in subsequent analyses. To explore which morphological variables better explain the variations in the functional setting, we ran *Generalized Linear Models* for the three different sets. This method allows us to model the morphology that enables a particular functional trait. Grasping was considered the only response variable, taking the value of 0 or 1, while the original variables retained by the PCAs were considered predictor variables. Our analyses yielded six variables associated with grasping abilities: two belong to the carpal bones, two belong to the metacarpals and two belong to the phalanges. Grasping in lizards can be performed with hands exhibiting at least two different independently originated combinations of bones. The first is a combination of a highly elongated centrale bone, reduced palmar sesamoid, divergence angles above 90°, and slender metacarpal V and phalanges, such as exhibited by *Anolis* sp. and *Tropidurus* sp. The second includes an elongated centrale bone, lack of a palmar sesamoid, divergence angles above 90°, and narrow metacarpal V and phalanges, as exhibited by geckos. Our data suggest that the morphological distinction between graspers and non-graspers is demonstrating the existence of ranges along the morphological continuum within which a new ability is generated. Our results support the hypothesis of the nested origin of grasping abilities within arboreality. Thus, the manifestation of grasping abilities as a response to locomotive selective pressure in the context of narrow-branch eco-spaces could also enable other grasping-dependent biological roles, such as prey handling.

## Introduction

The modern adaptive paradigm reasons that complex organic structures are mainly shaped by natural selection, establishing the premise that form and function are intimately related (Lauder, 1996). This theoretical framework emphasizes the importance of natural selection in altering phenotypes as a response to environmental demands (Ricklefs & Miles, 1994; Vanhooydonck & Irschick, 2002; Goodman et al., 2009; Houssaye et al., 2010; Fabre et al., 2013).

The tetrapod hand is one of the anatomical structures responsible for physical interaction between the environment and organisms. The hand represents such an iconic example accuracy of the form and function that (Darwin 1859, p. 434) himself was astonished:

“*What can be more curious than the hand of a man formed for grasping, that of a mole for digging, the leg of the horse, the paddle of the porpoise and the wing of the bat, should all be constructed on the same pattern and should include the same bones, in the same relative positions*?”

Function has been defined as the use or mechanical role of phenotypic features. The term is restricted to the action or property that a structure is able to exert (Ricklefs & Miles, 1994). The biological-role, implies the way in which that structure uses the environment throughout an organism’s life (Kardong, 2007; Ricklefs & Miles, 1994). In this sense grasping is defined as a mechanical role of an appendage (a hand, a tail, a foot) that exerts forces normal to the surface of the support on which the animal is situated or on an object that is held. These forces increase frictional resistance to slipping (Cartmill, 1985; Ricklefs & Miles, 1994; Sustaita et al., 2013). Thus, grasping is particularly versatile and effective at maintaining contact with the substratum and helping climbers avoid the risk of falling (biological role) (Hildebrand, 1995). In such a way grasping is one of a few functions that, in conjunction with hooking, arm swinging, flying, and adhering, allowed for incursion into the arboreal eco-space (Hildebrand & Goslow, 2001). Although these mechanisms are interpreted as the result of independent evolution it is possible to identify ecomorphological adaptive-patterns for similar arboreal behaviors (Fröbisch & Reisz, 2009). Of these motor functions, grasping is one of the more widespread and well-known (Hildebrand, 1995). The presence of a grasping hand in the human lineage has been repeatedly linked to the development of technology (Napier, 1956; Marzke, Wullstein & Viegas, 1992; Marzke, 1997; Susman, 1998; Kivell et al., 2011; Sustaita et al., 2013). Despite of its importance in delineating the evolutionary history of this function, grasping abilities have seldom been extensively considered out of the mammalian context (Gray, 1997; Manzano, Abdala & Herrel, 2008; Abdala et al., 2009; Mendyk & Horn, 2011; Sustaita et al., 2013).

Grasping hands probably originated in the first tetrapods (Iwaniuk & Whishaw, 2000), given that skilled manual abilities have been reported in amphibians (Gray, 1997; Manzano, Abdala & Herrel, 2008), crocodilians (Iwaniuk & Whishaw, 2000), Squamates (Abdala et al., 2009; Mendyk & Horn, 2011; Fontanarrosa & Abdala, 2014) and mammals (Costello & Fragaszy, 1988; Whishaw & Pellis, 1990; Godinot & Beard, 1991; Hamrick, 1996; Ivanco, Pellis & Whishaw, 1996; Iwaniuk & Whishaw, 2000; Sargis, 2001; Jungers et al., 2005; Lemelin et al., 2008; Kirk et al., 2008; Pouydebat et al., 2008; Tocheri et al., 2008; Toussaint & Meugnot, 2013; Fabre et al., 2013; among many others). There are at least two hypotheses regarding the origins of the grasping hand: (a) they originated in the context of the capture of mobile prey or hunting behavior (Gray, 1997; Iwaniuk & Whishaw, 2000; Godinot, 2007; Sustaita et al., 2013); (b) they derived from arboreal locomotion on thin branches. Species from taxa that exhibit manual grasping abilities also display pedal grasping abilities (Sustaita et al., 2013; Abdala et al., 2014), allowing the feet of arboreal animals to safely adhere to the narrow branches, thus freeing the forelimbs to perform other specialized functions (Brácha, Zhuravin & Bureš, 1990; Gray, 1997; Salesa et al., 2006; Endo et al., 2001; Manzano, Abdala & Herrel, 2008; Sustaita et al., 2013; Fabre et al., 2013; Herrel et al., 2013); Recently, Toussaint & Meugnot (2013) considered both aforementioned hypotheses when analyzing the grasping evolution of a small primate (see also Reghem et al., 2011). They conclude that the narrow branch niche may be an important selective pressure on the emergence of manual food grasping in primates (see also Sussman, 1991; Sussman & Raven, 1978), supporting previous inferences made for non-mammalian groups. Gray (1997), Manzano, Abdala & Herrel (2008), Abdala et al. (2009), and Sustaita et al. (2013) also conclude that grasping evolved only in arboreal groups subjected to narrow branch substrates, suggesting that the ability to climb is a preadaptation for the ability to grasp prey.

As an enhanced forelimb movement, grasping has been reported in at least six lizard lineages: Gekkota (Abdala et al., 2009), Polychrotids (Abdala et al., 2009), Dactyloids (Abdala et al., 2009), Tropidurids (Abdala et al., 2009), Chamaleonids (Higham & Anderson, 2013; Herrel et al., 2013; Diaz & Trainor, 2015), and Varanids (Mendyk & Horn, 2011). Studies of grasping as an enhanced manual ability in non-mammalian tetrapods have focused on the anatomy of muscle and tendon structure (Manzano, Abdala & Herrel, 2008; Abdala et al., 2009; Sustaita et al., 2013), biomechanics (Manzano, Abdala & Herrel, 2008; Abdala et al., 2009) and behavior (Gray, 1997; Mendyk & Horn, 2011). Evolutionary schemes of the skeletal patterns linked to this ability that include non-mammalian tetrapods are scarce (Fontanarrosa & Abdala, 2014). Fontanarrosa & Abdala (2014) conducted a qualitative analysis of the bone anatomy of various hand grasping squamate species, the evidence of which aligned with previous studies that also show that grasping capability has clear osteological consequences (Hamrick, 1996; Hopson, 2001; Bloch & Boyer, 2002; Kirk et al., 2008; Salton & Sargis, 2008; Fröbisch & Reisz, 2009; Fabre et al., 2013; Feix et al., 2015). The grasping abilities associated with lizards correspond to the “power grip”, as defined by Napier (1956): “The object may be held in a clamp formed by the partly flexed fingers and the palm, counter pressure being applied by the thumb lying more or less in the plane of the palm”.

The lizard hand skeleton is comprised of about 28 specialized bones. It includes, from the proximal to the distal region, the carpus, the metacarpus and a series of phalanges that composes each digit (Romer, 1956; Flower, 1885; Renous-Lécuru, 1973). Additionally, in most lizards the palmar sesamoid covers the ventral surface of the carpus (Jerez, Mangione & Abdala, 2009; Otero & Hoyos, 2013; Fontanarrosa & Abdala, 2014).

The objective of this work is to ascertain the existence of morphometric descriptors in the hand skeleton of lizards related to grasping functionality. The identified morphometric patterns are examined within the context of the most accepted phylogenetic hypothesis of Squamata in order to assess whether determined morphological traits of the hand skeleton of lizards can be interpreted as adaptations for grasping. The identification of anatomical features directly associated with grasping capabilities establishes a basis for ecomorphological interpretations of the fossil record.

Previous studies regarding skeletal hand patterns and their relationship with grasping highlighted the importance of the centrale bone, the palmar sesamoid, the divergence angle of the first digits, and metacarpal length in relation to grasping capabilities. The centrale bone is linked to an increased hand or wrist versatility (Thorington & Darrow, 2000; Abdala et al., 2009; Sustaita et al., 2013; Fontanarrosa & Abdala, 2014), and thus we predict that the narrower centrale present in grasping hands plays a significant role in the expression of these grasping capabilities. The palmar sesamoid, however, has been linked to an impaired grasping ability (Abdala et al., 2009; Fontanarrosa & Abdala, 2014), and we therefore predict that it will be reduced or absent in grasping hands. The wide divergence of the first digits characterize grasping tetrapod hands (Napier, 1956; Youlatos, 1999; Lemelin & Schmitt, 1998; Endo et al., 2001; Sheil & Alamillo, 2005; Manzano, Abdala & Herrel, 2008), thus we predict that lizard grasping hands will also exhibit greater divergence angles between digits. Arboreal tetrapods have short metapodium and long phalanges (Lemelin & Jungers, 2007; Fröbisch & Reisz, 2009), therefore we predict that the metapodial of lizard grasping hands will display short metacarpals and elongated phalanges. Finally, taking into account the pervasive link between arboreality and the origin of grasping abilities (Sussman, 1991; Fabre et al., 2013; Sustaita et al., 2013), we predict that grasping as a character will be nested within arboreality.

## Materials and Methods

### Morphometric variables

Measurements were made on skeletons that had been cleared and double stained with Alcian Blue and Alizarin Red, following the methodology presented in Wassersurg (1976). Photographs were taken with a stereo dissecting microscope (Nikon, SMZ-10, Nikon Corp., Tokyo, Japan).

**Figure 1:**
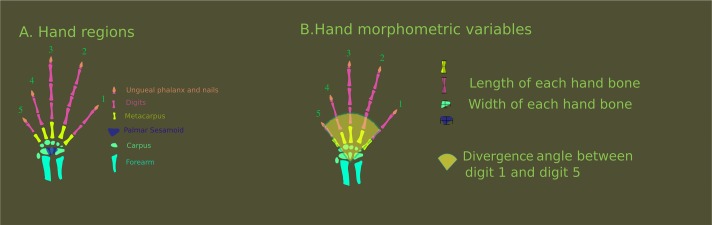
Hand regions and morphometric variables. (A) Hand regions and forearm bones. (B) Morphometric variables used in the morphometric matrix. Vertical vectors symbolize the length of each hand bone, horizontal vectors symbolize the width of each hand bone. Fan shaped draw symbolize the divergence angle between digit one and five. Note that although the figure shows only one example of measurements for each hand region, the same logic was applied for each bone of the hand. An exception was made for ungueal phalanx: they were not measured due to the ambiguous distal edges.

A morphological matrix was constructed (Online Resource I) using 51 morphometric variables (Fig. 1) in 278 specimens, from 24 genera and 13 families of Squamata, following two main criteria: (1) maximization of the morphological range sampled; and (2) maximization of the phylogenetic representation (taking into account only those squamate lineages with developed limbs). Subsets of the matrix that corresponded to functional modules, specifically the carpals, metacarpals and phalanges, were disaggregated for a more focused analysis. The morphometric variables were processed using ImageJ 1.43 (US National Institutes of Health, Bethesda, MD, USA). Institutional approval for this research is given with the financial support.

### Functional settings

**Figure 2:**
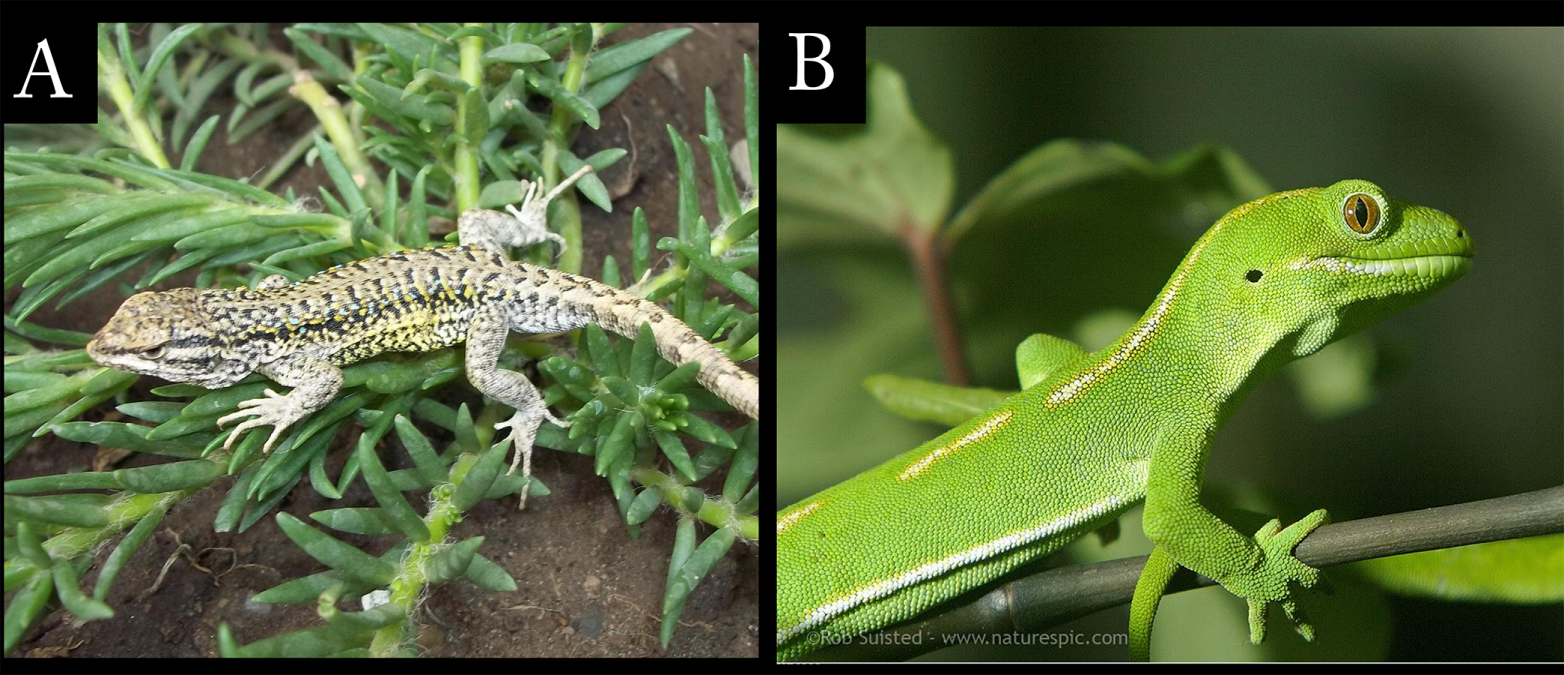
Functional Setting. Functionality was defined as either prehensile or non-prehensile according to Napier (1956). (A) Non-prehensile example: *Liolaemus ruibali* (Argentina) with its left hand resting over a branch. Notice that it does not grasp the branch. Photo: Marcos Paz. (B) Prehensile example: *Naultinus elegans* (New Zealand) grasping a branch with its right hand. Notice that the second cleft surrounds the branch. Photo: Rob Suisted.

Functionality was defined as either prehensile or non-prehensile. Species were assigned to each category according to Abdala et al. (2009), Sustaita et al. (2013), Fontanarrosa & Abdala (2014) and personal observations (Fig. 2; Table 1).

**Table 1:** Manual capabilities assignment. Manual capabilities were assigned based on: literature data (#), personal observations (+), inferred from their habitat use and photographs (°).

Genus	Grasping	Source
Ameiva	0	Fontanarrosa & Abdala (2014) #
Ameivula	0	Arias et al. (2014)
Anisolepis	?	–
Anolis	1	Abdala et al. (2009) #, Fontanarrosa & Abdala (2014); Gabriela Fontanarrrosa +
Cercosaura	0	Fontanarrosa & Abdala (2014)
Cnemidophorus	0	Virginia Abdala +, Gabriela Fontanarrrosa +
Eublepharis	1	Fontanarrosa & Abdala (2014)
Thecadactylus	1	Fontanarrosa & Abdala (2014); Gabriela Fontanarrrosa +
Homonota	1	Fontanarrosa & Abdala (2014); Virginia Abdala +, Gabriela Fontanarrrosa +
Iguana	0	Tulli et al. (2009); Virginia Abdala +, Gabriela Fontanarrrosa +
Kentropix	0	Virginia Abdala +
Liolaemus	0	Tulli et al. (2009) #; Fontanarrosa & Abdala (2014) # Halloy, Etheridge & Burghardt (1998) #; Virginia Abdala +, Gabriela Fontanarrrosa +
Mabuya	0	Fontanarrosa & Abdala (2014) #
Pholidobolus	0	Torres Carvajal et al. (2014) (°)
Phyllopezus	1	Fontanarrosa & Abdala (2014) #, Virginia Abdala +
Gonatodes	1	Gabriela Fontanarrosa +
Phymaturus	0	Lobo & Quinteros (2005) # Virginia Abdala +
Physignathus	0	Honda et al. (2000)
Prionodactylus	0	Vitt et al. (2003) (°)
Proctoporus	0	Mamani, Goicoechea & Chaparro (2015) #
Stenocercus	0	Frost (1992) (#), (°)
Tropidurus	1	Virginia Abdala +
Uromastix	0	Wilms et al. (2007) #
Varanus	0	Mendyk & Horn (2011) #

**Notes.**

0, absent; 1, present; ?, unknown

### Statistical analysis

To reduce the dimensions of the dataset and to simplify the organization of variables, three PCAs (Principal Component Analyses) were performed using the subsets of (1) carpal variables, (2) metacarpal variables, and (3) phalanges variables. All variables were size-corrected by the geometric mean (each species measurement divided by the nth root of the product of values of a species’ vector of n variables) (Morales & Giannini, 2010). Based on the PCA results, we ordered the original variables in relation to their loadings (absolute values). Each loading represents the correlation between the original variable and the linear combination of the original variables (PCs). The PCs were retained based on the graphical Print Method which describes the relative importance (its variance) of each axis and displays up to which PC the variance is significant. The variables that had the most significant contributions to the construction of the synthetic variables (Table 2) were retained to be used in subsequent analyses (GLM).

**Table 2:** Table of ordered variable loadings. Each value represents the correlation between the original variable and the linear combination of the original variables (PCs). The loadings were ordered by their absolute values.

Ordered variables	Correlation with PC 1	Ordered variables	Correlation with PC 2
**CARPAL PCA**			
Radiale, width	−0.85	Pisiform, proximal-distal distance	0.61
Ulnare, proximal-distal distance	0.27	Pisiform, width	0.50
Centrale, proximal-distal distance	0.27	Ulnare, width	−0.32
Radiale, proximal-distal distance	0.23	Distal carpal 4, proximal-distal distance	−0.30
Distal carpal 4, proximal-distal distance	0.15	Radiale, width	−0.22
Centrale, width	−0.13	Centrale, proximal-distal distance	−0.21
Distal carpal 5, proximal-distal distance	0.12	Radiale, proximal-distal distance	−0.18
Pisiform, width	−0.09	Distal carpal 1, proximal-distal distance	0.12
Distal carpal 2, proximal-distal distance	0.08	Distal carpal 2, proximal-distal distance	−0.11
Ulnare, width	−0.07	Distal carpal 5, proximal-distal distance	−0.11
Distal carpal 1, width	−0.07	Distal carpal 1, width	−0.06
Distal carpal 4, width	0.07	Distal carpal 2, width	0.06
Distal carpal 2, width	−0.02	Distal carpal 3, proximal-distal distance	−0.05
Distal carpal 3, proximal-distal distance	−0.02	Distal carpal 3, width	0.04
Distal carpal 3, width	−0.02	Distal carpal 4, width	0.04
Pisiform, proximal-distal distance	0.02	Distal carpal 5, width	−0.04
Distal carpal 1, proximal-distal distance	−0.02	Ulnare, proximal-distal distance	−0.03
Distal carpal 5, width	−0.01	Centrale, width	0.01
**Proportion of Variance**	**0.24**		**0.14**
**METARCARPAL PCA**			
Metarcarpal 3 Length	−0.54		
Metarcarpal 4 Length	−0.49		
Metarcarpal 2 Length	−0.48		
Metarcarpal 1 Length	−0.35		
Metarcarpal 5 Length	−0.32		
Metarcarpal 4 Width	−0.06		
Metarcarpal 5 Width	−0.06		
Metarcarpal 3 Width	−0.05		
Metarcarpal 1 Width	−0.05		
Metarcarpal 2 Width	−0.05		
**Proportion of Variance**	**0.97**		
**DIGITAL PCA**			
Digit 5, Phalanx 2, Length	−0.42	Digit 3, Phalanx 1, Length	−0.38
Digit 4, Phalanx 4, Length	−0.39	Digit 1, Phalanx 1, Length	0.36
Digit 3, Phalanx 3, Length	−0.33	Digit 4, Phalanx 2, Length	−0.36
Digit 4, Phalanx 3, Length	−0.30	Digit 4, Phalanx 4, Length	0.35
Digit 4, Phalanx 1, Length	−0.29	Digit 3, Phalanx 2, Length	−0.34
Digit 5, Phalanx 1, Length	−0.29	Digit 2, Phalanx 2, Length	0.28
Digit 2, Phalanx 2, Length	−0.26	Digit 3, Phalanx 3, Length	0.25
Digit 3, Phalanx 2, Length	−0.26	Digit 4, Phalanx 3, Length	−0.24
Digit 1, Phalanx 1, Length	−0.25	Digit 4, Phalanx 1, Length	−0.24
Digit 3, Phalanx 1, Length	−0.21	Digit 5, Phalanx 1, Length	−0.23
Digit 2, Phalanx 1, Length	−0.16	Digit 2, Phalanx 1, Length	−0.11
Digit 4, Phalanx 3, Width	0.07	Digit 1, Phalanx 1, Width	−0.08
Digit 3, Phalanx 2, Width	0.07	Digit 5, Phalanx 2, Length	0.08
Digit 4, Phalanx 1, Width	0.07	Digit 4, Phalanx 2, Width	0.07
Digit 4, Phalanx 4, Width	0.06	Digit 4, Phalanx 3, Width	0.06
Digit 5, Phalanx 1, Width	0.06	Digit 4, Phalanx 1, Width	0.05
Digit 3, Phalanx 1, Width	0.06	Digit 4, Phalanx 4, Width	0.04
Digit 2, Phalanx 2, Width	0.05	Digit 3, Phalanx 1, Width	0.04
Digit 3, Phalanx 3, Width	0.05	Digit 5, Phalanx 1, Width	0.04
Digit 5, Phalanx 2, Width	0.04	Digit 2, Phalanx 1, Width	−0.04
Digit 4, Phalanx 2, Width	0.04	Digit 3, Phalanx 2, Width	0.03
Digit 2, Phalanx 1, Width	0.04	Digit 3, Phalanx 3, Width	0.02
Digit 4, Phalanx 2, Length	−0.03	Digit 5, Phalanx 2, Width	−0.01
Digit 1, Phalanx 1, Width	0.03	Digit 2, Phalanx 2, Width	0.01
**Proportion of Variance**	**0.55**		**0.10**

**Table 3:** Coefficient tables of the best models. Each box shows the model, the coefficients for each variable, their *p*-value and the Akaike Information Criterion value of the model. (A) Best fit model for the carpal subset, showing that gras ping can be considered as a function of centrale bone width and palmar sesamoid length because the slopes of each variables of the GLM are statistically significant. Additionally, with this model we obtained the lesser AIC value. (B) Best fit model for the metacarpal subset, showing that grasping can be considered as a function of the first metacarpal width and the divergence angle between digit one and digit five. (C) Best fit model for the digital subset showing that grasping can be considered as a function of the fourth phalanx width of digit four and the first phalanx length of digit one.

Predictor variable	Slope	*p*-value
(A) **Carpal model**		
Centrale bone width	−**14.165**	1.67e−07 (^∗∗∗^)
Palmar sesamoid length	−**2.674**	3.11e−05 (^∗∗∗^)
**Akaike Information Criterion:** 121.49		
(B) **Metacarpal model**		
First metacarpal width	−**20.1**	1.44e−05 (^∗∗∗^)
Divergence anlge between digit 1 and digit 5.	**0.11**	5.91e−07 (^∗∗∗^)
**Akaike Information Criterion:** 104.51		
(C) **Digital model**		
Fourth phalanx width of digit 4	**10.3**	2.66e−05 (^∗∗∗^)
First phalanx length of digit 1	**7.2**	1.14e−11 (^∗∗∗^)
**Akaike Information Criterion:** 100.57		

**Notes.**

Significance codes (*) shows the signficance level below which is located the *p*-value. *α* = 0 (***), *α* = 0.001 (**), *α* = 0.01 (*).

To explore which morphological variables retained by the PCAs (Table 2) better explain the variations in the functional setting, we ran *Generalized Linear Models* (**GLMs;** Table 3) for the three different sets, with a binomial error structure for non-aggregate data. The GLM (Nelder & Wedderburn, 1972) represent a modification of the ordinary linear regression that can be used when the response variables have error distribution models that differ from a normal distribution. They allow data to be related to the response variables by a link function depending on the distribution family of the error. This method allows us to model the morphology of a particular functional trait. Grasping was considered the only response variable and takes a value of 0 or 1, while all the original variables retained by the PCAs (Table 2) were considered as predictor variables. The models were performed using the canonical link function for a *Bernoullli* distribution. The relation between each selected predictor variable was first assessed independently, while the other significantly contributing variables were then added sequentially to the model in a forward process (not automatic). To define which model best fit the data, we followed the Akaike Information Criterion (AIC) (Burnham & Anderson, 2004). Throughout the process, variables that were determined not to be good predictors of grasping function were deleted. All the statistical analyses were implemented in the R statistical environment (R Development Core Team, 2011).

Morphological traits that were linked by the model to grasping were then mapped into the selected phylogeny.

### Character mapping

Based on the GLMs, the variables included in the minimal adequate model (MAM) were treated as continuous characters to trace their evolutionary history onto a selected phylogeny of Squamata using Mesquite (Maddison & Maddison, 2009). Additionally, two discrete variables were considered, grasping and arboreality. The method assigns values to the ancestral nodes allowing for a formal evaluation of the evolutionary history of the selected characters.

The chosen cladogram is based on molecular characters (Wiens et al., 2012) and was reduced to display only the taxa used in this analysis. Unfortunately, as most of the species studied in this work were not considered in the selected (or any other) molecular cladogram, inferences had to be made based on the genera. We recognize that this is a shortcoming of the study, but we are confident that most of the optimizations would be supported in a more adequate phylogeny.

## Results

Three models that best fit the data were obtained, one model for each anatomical subset: carpal, metacarpal, and digits.

**Figure 3:**
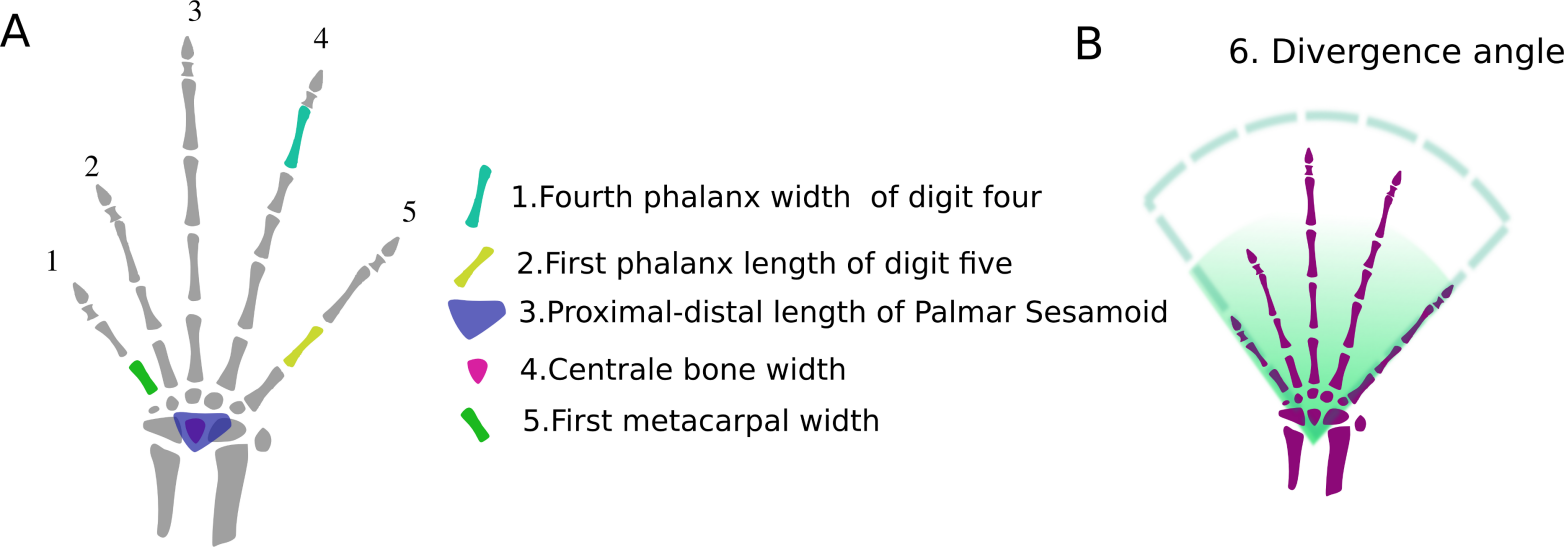
Variables that best fit the data after performing the GLMs. (A) Hand dimension variables: the skeletal structures that correspond to those variables are highlighted. Carpus: (1) Width of the centrale bone; (2) Proximal-distal length of the palmar sesamoid; Metacarpus : (3) Width of the first metacarpal; Digits : (4) Length of the first phalanx of digit five; (4) Width of the fourth phalanx of digit four. (B) Dispositional variable: divergence angle between the first metacarpal and the fifth one.

Six variables (Fig. 3, Table 3) were retained in the models. The carpal model indicates that grasping is a functional output of the width of the centrale bone and the proximal-distal length of the palmar sesamoid. The metacarpal model indicates that grasping is a functional output of the width of the first metacarpal and the divergence angle between digits one and five. The digit model indicates that grasping is a functional output of the length of the first phalanx of digit five and the width of the fourth phalanx of digit four.

**Figure 4:**
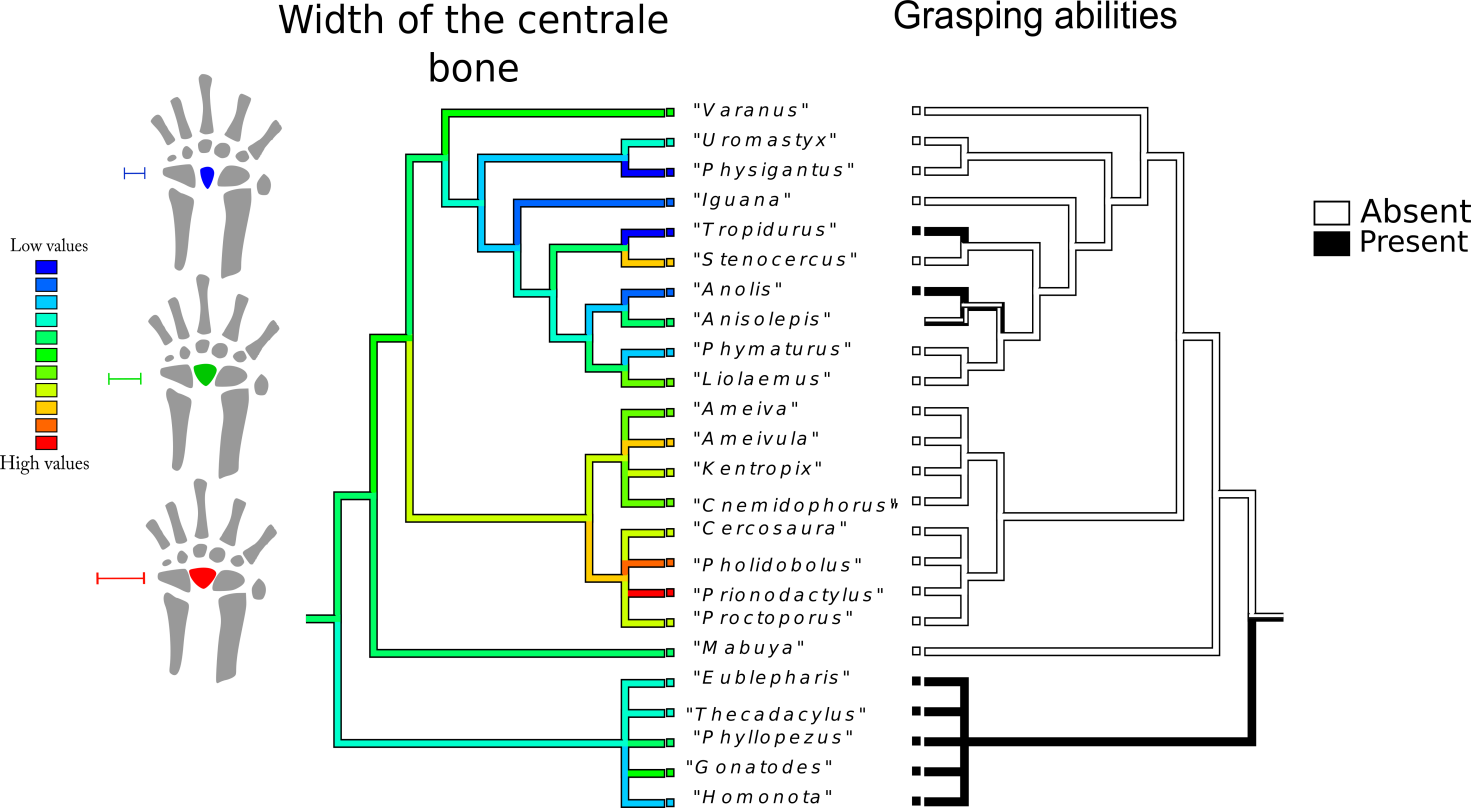
Character history of the width of the centrale bone compared to the character history of grasping abilities. Left tree: character history of the width of the centrale bone. The most parsimonious state for the common ancestor is an intermediate value (green range). The narrowest centrale bone appears independently in *Physignatus* sp. and *Tropidurus* sp. (extreme blue on the color gradient), followed by *Anolis* sp. and *Iguana* sp. Gekkota also tend to exhibit narrow centrale bones, although not the narrowest. Gymnophthalmidae and Teiidae show a trend of intermediate to high values; including *Prionodactylus* sp. that has the widest centrale bone in the phylogenetic tree (extreme red on the color gradient). Anguimorpha and Iguania show a trend toward narrow centrale bones, with the exception of *Stenocercus* sp., which has a wider centrale. Right tree: character history of grasping abilities. The most parsimonious state for the common ancestor is ambiguous. This character shows three independent origins in the tree: *Tropidurus* sp., *Anolis* sp., and Gekkota. In this last case, grasping ability is a synapomorphy of the group.

### Character mapping

The evolutionary history of the width of the centrale bone (Fig. 4): The most parsimonious state for the common ancestor is an intermediate value. The narrowest centrale bone appears independently in *Physignatus* sp. and *Tropidurus* sp., followed by *Anolis* sp. and *Iguana* sp. Gekkota also tend to exhibit narrow centrale bones, although not the narrowest. Gymnophthalmidae and Teiidae show a trend of intermediate to high values; including *Prionodactylus* sp. that has the widest centrale bone in the phylogenetic tree. Anguimorpha and Iguania show a trend toward narrow centrale bones, with the exception of *Stenocercus* sp., which has a wider centrale.

**Figure 5:**
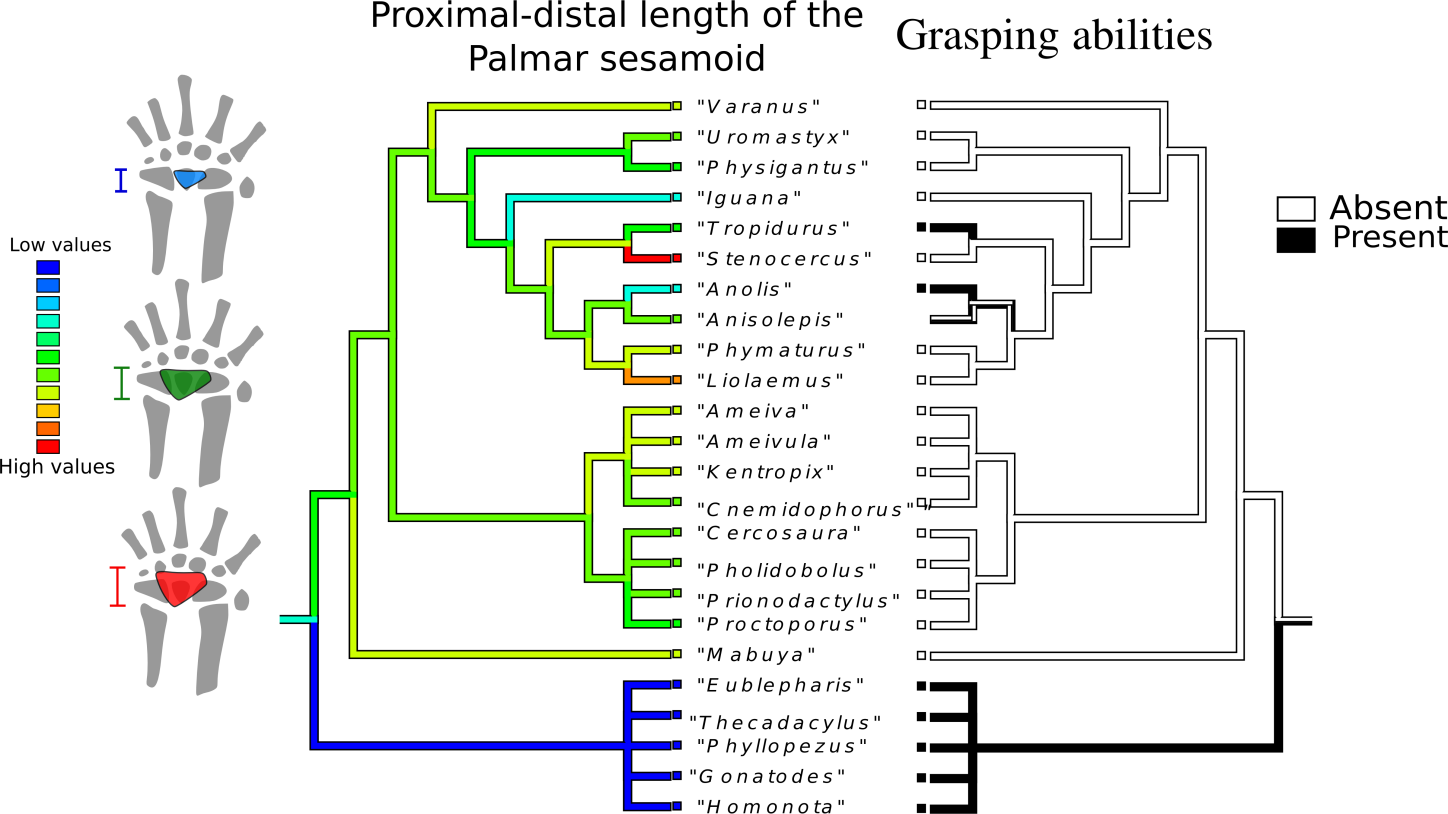
Character history of the proximal-distal length of the palmar sesamoid compared to the character history of grasping abilities. Character history of the proximal-distal length of the palmar sesam oid compared to the character history of grasping abilities Left tree: character history of the proximal-distal length of the palmar sesamoid. The most parsimonious state for the common ancestor is a low value (blue range), although the most frequent states in the tree are intermediate values (green range). Gekkota lack a palmar sesamoid. Gymnophthalmidae and Teiidae tend to have intermediate values, as well as Anguimorpha and Iguania, with the notable exception of *Stenocercus* sp. and *Liolaemus* sp., which possess the biggest palmar sesamoid (red branch). Right tree: character history of grasping abilities.

Proximal-distal length of the palmar sesamoid (Fig. 5): The most parsimonious state for the common ancestor is a low value (light blue range), although the most frequent states in the tree are intermediate values (green range). Loss of the palmar sesamoid is a synapomorphy of Gekkota. Gymnophthalmidae and Teiidae tend to have intermediate values, as do Anguimorpha and Iguania, with the notable exception of *Stenocercus* sp. and *Liolaemus* sp., which possess the biggest palmar sesamoid.

**Figure 6:**
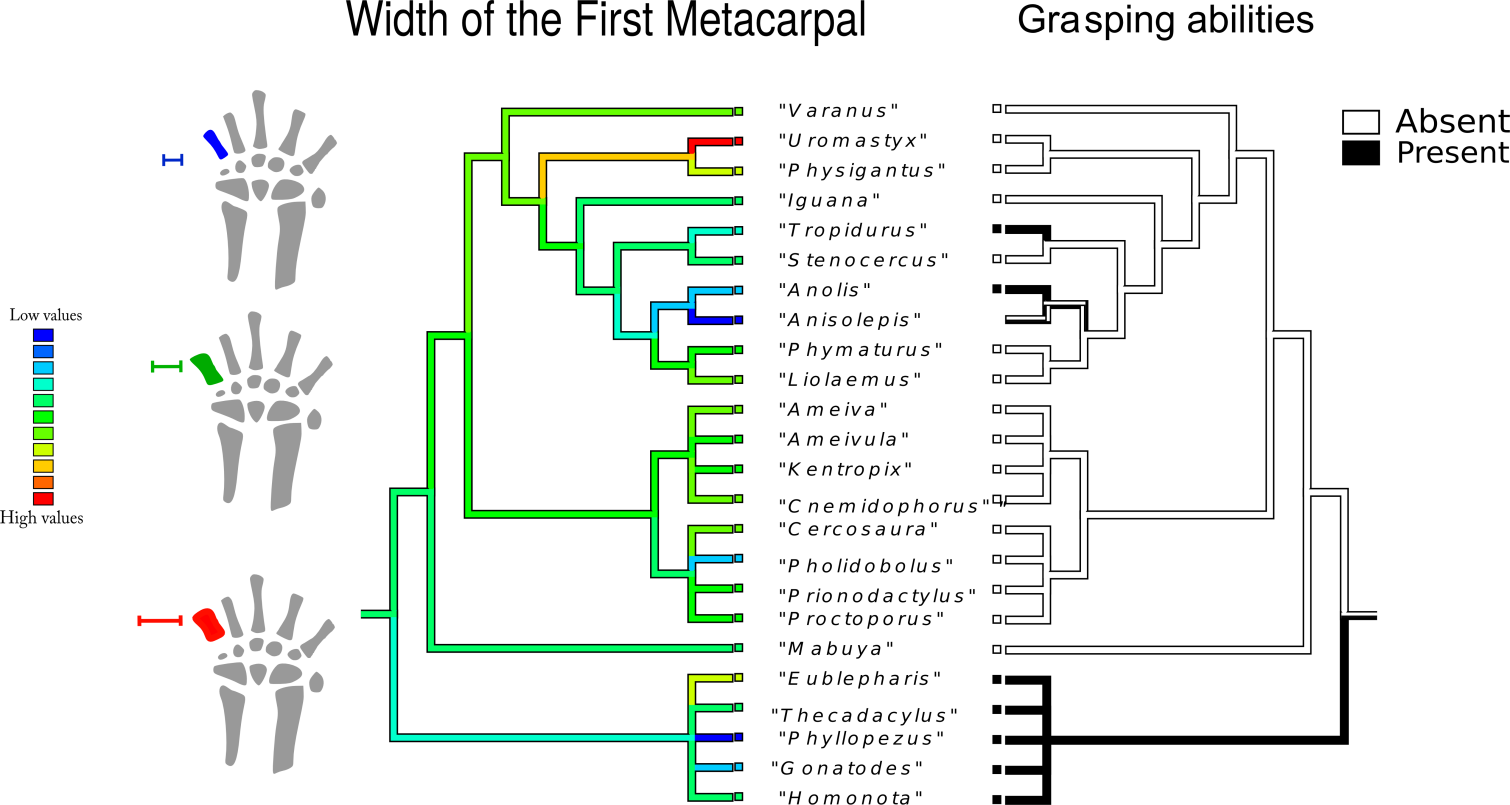
Character history of the width of the first metacarpal compared to the character history of grasping abilities. Left tree: character history of the width of the first metacarpal. The most parsimonious state for the common ancestor is an intermediate value, which is also coincidently the most frequent value in the tree (green range). The narrowest first metacarpal bone, which appears independently in *Phyllopezus* sp. and *Anisolepis* sp., is followed by *Anolis* sp., *Gonatodes* sp., and *Pholidobolus* sp. Gekkota tend to present narrow first metacarpal bones. Gymnophthalmidae and Teiidae show a trend toward first metacarpals with intermediate to wide values. Anguimorpha and Iguania show a wider range of first metacarpal widths, including representatives of the narrowest (*Anisolepis* sp.) and the widest (*Uromastix* sp. and *Physignatus* sp.) bones. Right tree: character history of grasping abilities.

Width of the first metacarpal (Fig. 6): the most parsimonious state for the common ancestor is an intermediate value, which is also coincidentally the most frequent value in the tree (green range). The narrowest first metacarpal bone, which appears independently in *Phyllopezus* sp. and *Anisolepis* sp., is followed by *Anolis* sp., *Gonatodes* sp., and *Pholidobolus* sp. Gekkota tend to present narrow first metacarpal bones, with the exception of *Eublepharis* sp., which has a higher value. Gymnophthalmidae and Teiidae demonstrate a trend toward first metacarpals with intermediate values. Anguimorpha and Iguania show a wider range of first metacarpal widths, including representatives of the narrowest (*Anisolepis* sp.) and the widest (*Uromastix* sp. and *Physignatus* sp.) bones.

**Figure 7:**
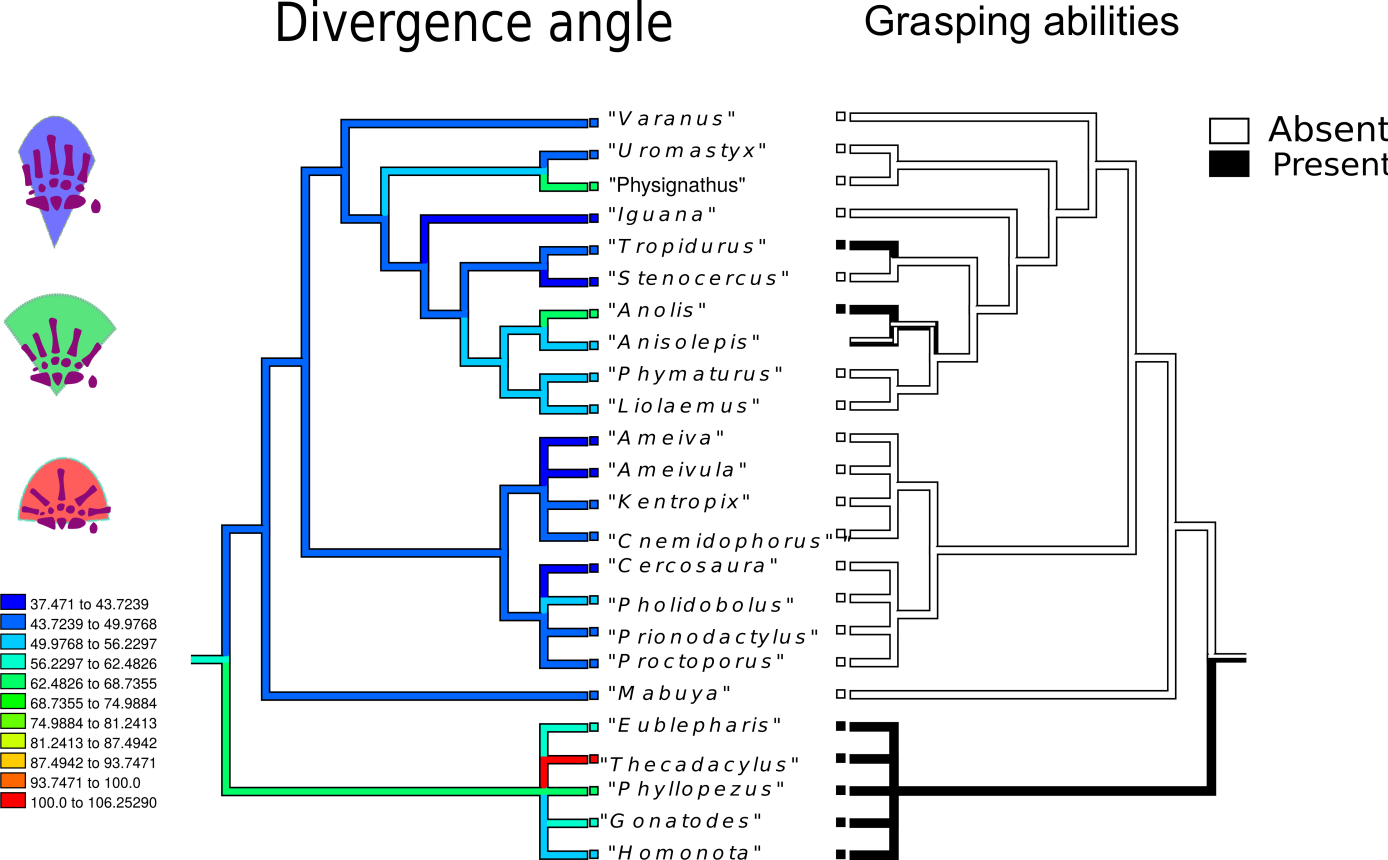
Character history of the divergenceangle compared to the character history of grasping abilities. Left tree: character history of the divergence angle between the first and the fifth metacarpals. The most parsimonious state for the common ancestor is 84°, but the most frequently encountered state corresponds to lower angles (blue range). The evolution of a higher divergence angle initiated in the branch that gave rise to Gekkota (average 100°). Angles over 90° appear independently in *Anolis* sp., Gekkota and *Physignatus* sp. In Gymnophthalmidae and Teiidae the general trend is to have angles smaller than 70°, including extreme values such as 40°in *Ameivula* sp. and *Cercosaura* sp. Anguimorpha and Iguania present a wider range of angles, from *Stenocercus* sp. with 44° to *Anolis* sp. and *Physignatus* sp., both between 70 and 81°. Right tree: character history of grasping abilities.

Divergence angle (Fig. 7): the most parsimonious state for the common ancestor is 84°, but the most frequently encountered state corresponds to lower angles (blue range). The evolution of a higher divergence angle initiates in the branch that gave rise to Gekkota (average 100°). Angles over 90° appear independently in *Anolis* sp., Gekkota and *Physignatus* sp. In Gymnophthalmidae and Teiidae the general trend is to have angles smaller than 70°, including extreme values such as 40° in *Ameivula* sp. and *Cercosaura* sp. Anguimorpha and Iguania present a wider range of angles, from *Stenocercus* sp. with 44° to *Anolis* sp. and *Physignatus* sp., both with 97°.

**Figure 8:**
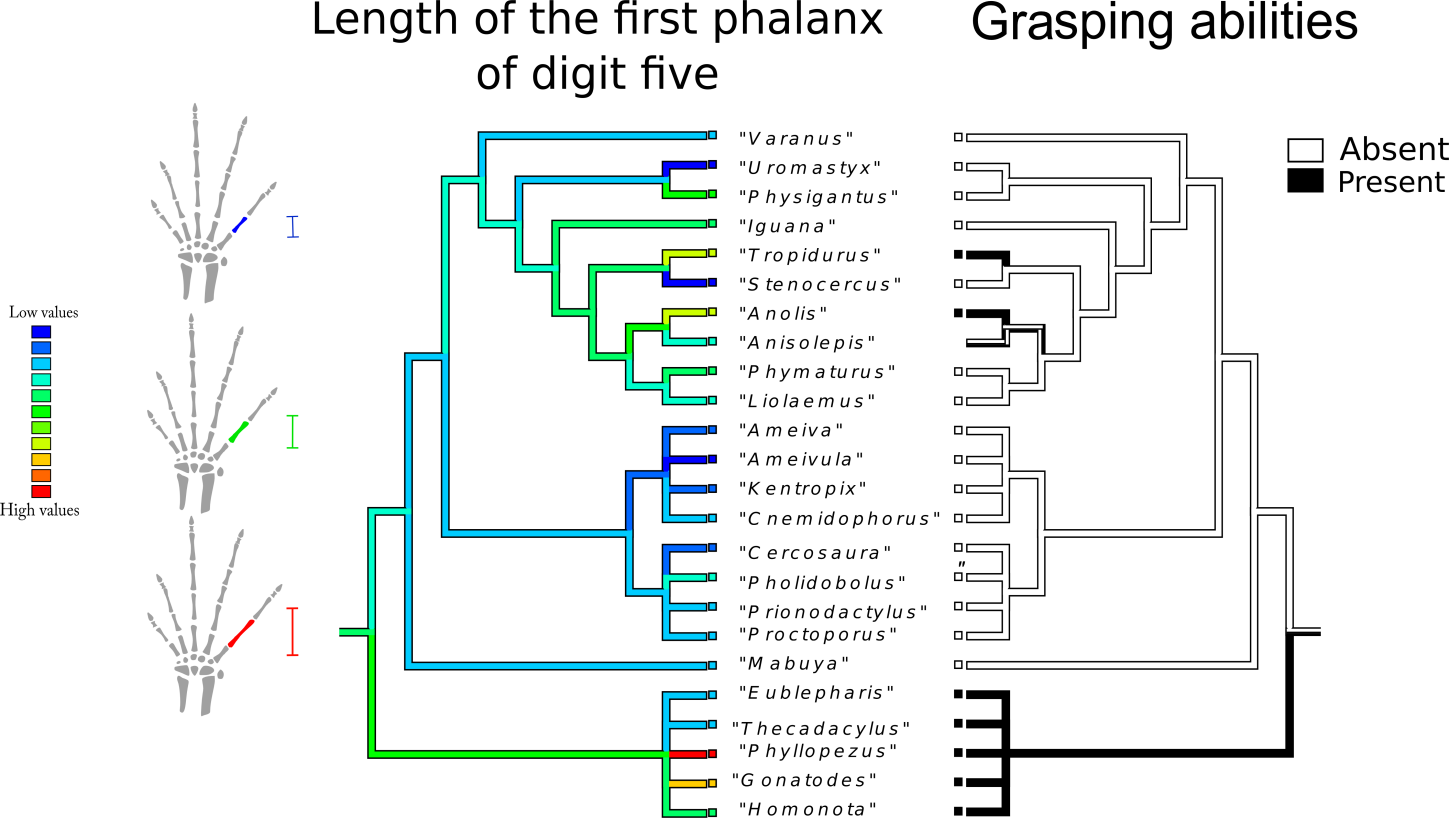
Character history of the length of the first phalanx of digit five compared to the character history of grasping abilities. Left tree: character history of the length of first phalanx of digit five. The most parsimonious state for the common ancestor is an intermediate value, whereas the most frequent state corresponds to low values (blue range). The longest first phalanx studied belongs to *Phyllopezus* sp. and *Gonatodes* sp. High values of this variable were also independently reached in *Anolis* sp., and *Tropidurus* sp., while low values were independently acquired by *Uromastix* sp., *Stenocercus* sp., and *Ameivula* sp. Gekkota displays a wide range of values. Gymnophthalmidae and especially Teiidae, tend to exhibit high values, whereas Anguimorpha and Iguania manifest a wider range. Right tree: character history of grasping abilities.

Length of first phalanx of digit five (Fig. 8): The most parsimonious state for the common ancestor is an intermediate value, whereas the most frequent state corresponds to low values (blue range). The longest first studied phalanx belongs to *Phyllopezus* sp. and *Gonatodes* sp. The high values of this variable were also independently reached in *Anolis* sp., and *Tropidurus* sp., while a shorter structure was independently acquired by *Uromastix* sp., *Stenocercus* sp. and *Ameivula* sp. Gekkota displays a wide range of values. Gymnophthalmidae and especially Teiidae, tend to exhibit low values, whereas Anguimorpha and Iguania present wider ranges.

**Figure 9:**
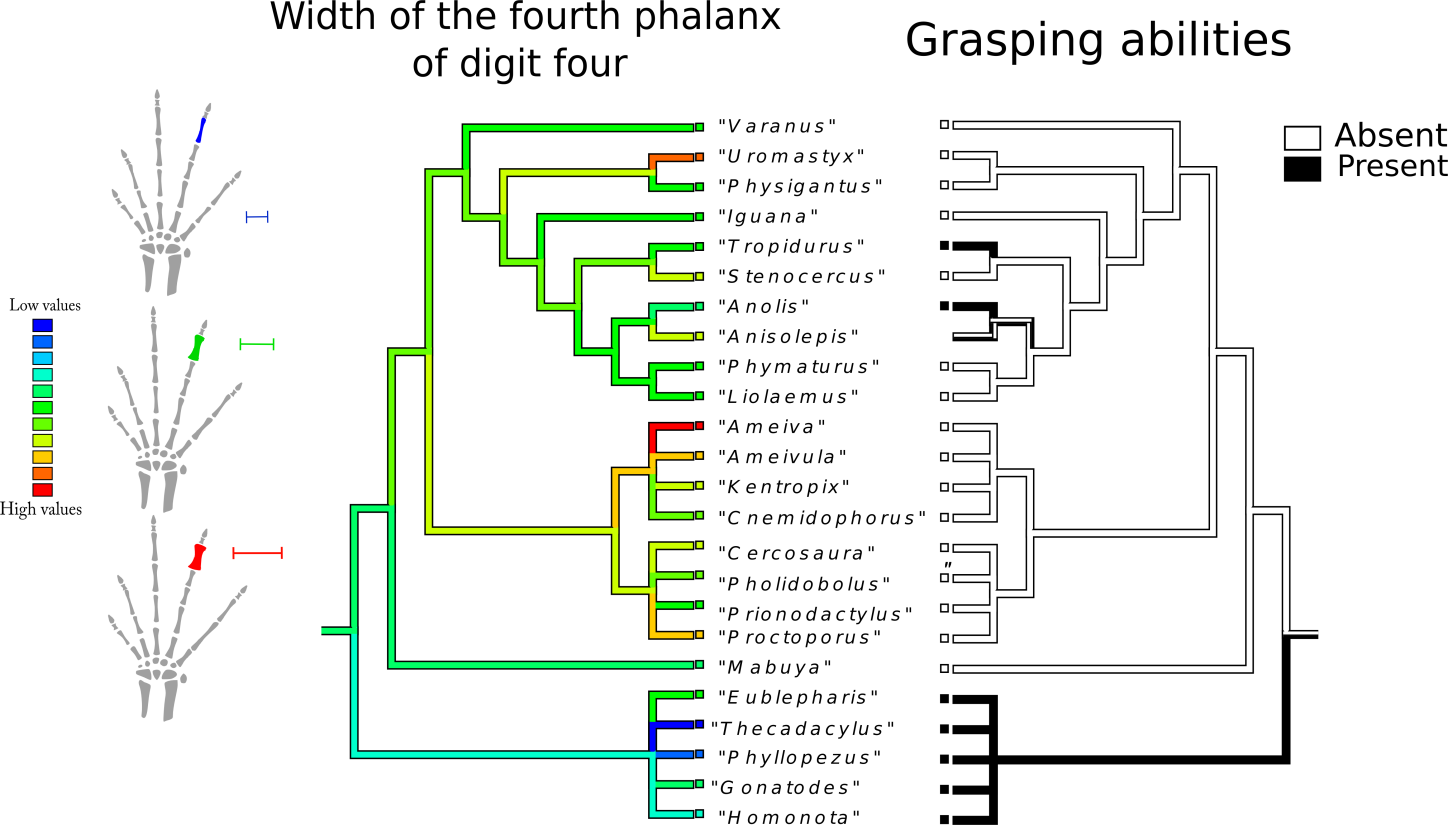
Character history of the width of the fourth phalanx of digit four compared to the character history of grasping abilities. Left tree: character history of the width of the fourth phalanx of digit four. The plesiomorphic and most frequent states in the tree are intermediate values (green range). The widest phalanx was independently acquired in *Ameiva* sp., *Uromastix* sp., *Ameivula* sp., and *Proctoporus* sp. The narrowing of the fourth phalanges began in the branch that gave rise to Gekkota, with *Phyllopezus* sp. and *Thechadactylus* sp. displaying the maximum expression of this process. Gymnophthalmidae and Teiidae show a wide range of values. Right tree: character history of grasping abilities.

Width of the fourth phalanx of digit four (Fig. 9): the plesiomorphic and most frequent state in the tree are intermediate values (green range). The widest phalanx was independently acquired in *Ameiva* sp., *Uromastix* sp., *Ameivula* sp. and *Proctoporus* sp. The thinning of the fourth phalanx began in the branch that gave rise to Gekkota, with *Phyllopezus* sp. and *Thechadactylus* sp. displaying the maximum expression of this process. Gymnophthalmidae and Teiidae show a wide range of values.

**Figure 10:**
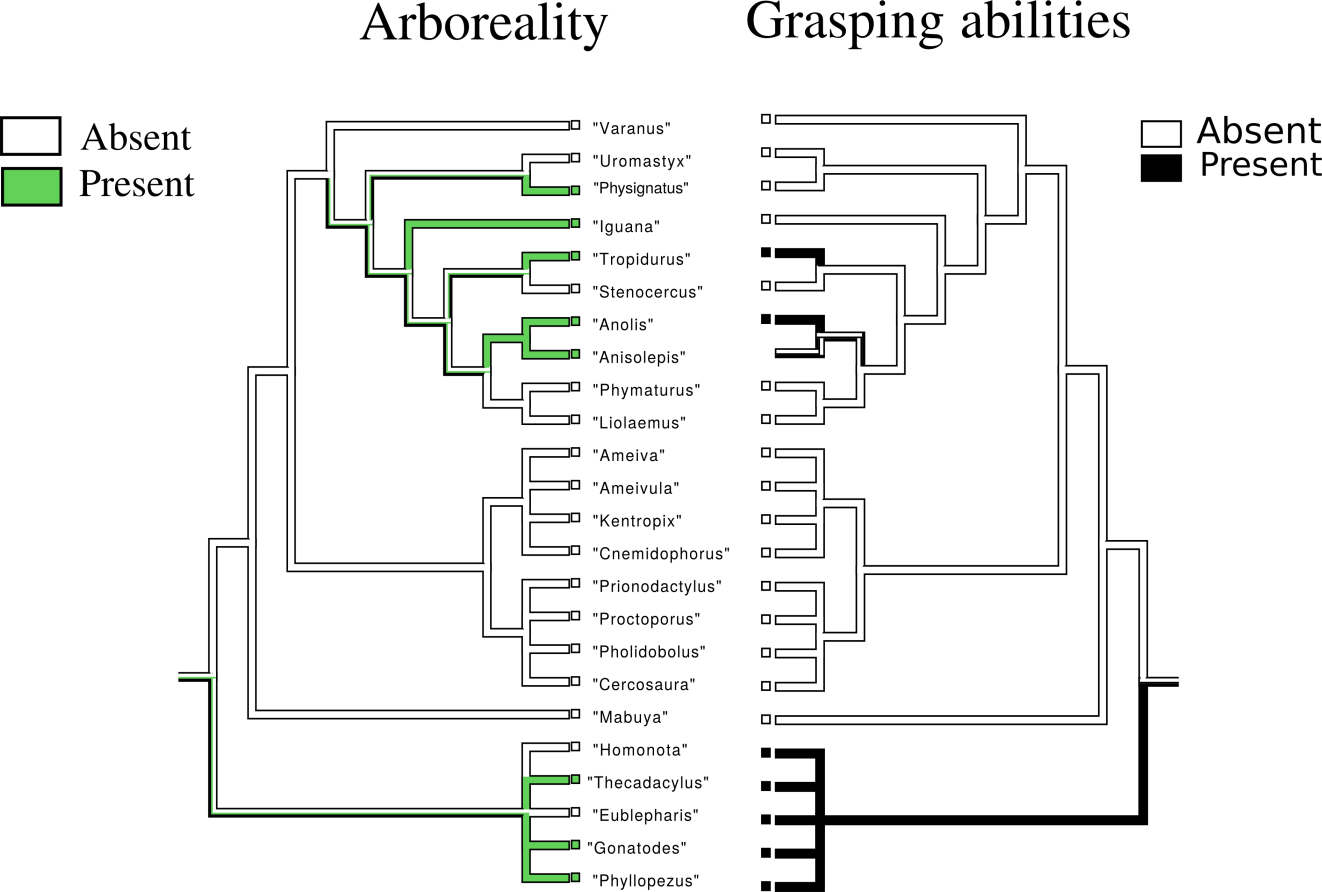
Character history of arboreality compared to the character history of grasping abilities. Left tree: character history of arboreality. The most parsimonious state for the common ancestor is ambiguous. Arboreality arose in two main nodes of the tree. One including *Physignatus* sp., *Iguana* sp., *Tropidurus* sp., *Anolis* sp., *Anisolepis* sp., and the other composed by Gekkota. Our data prevent us to asses whether they were independent origins. Right tree: character history of grasping abilities.

Arboreality (Fig. 10): the most parsimonious state for the common ancestor is ambiguous. The character appear in two main nodes of the tree, one including *Physignatus* sp., *Iguana* sp., *Tropidurus* sp., *Anolis* sp., and *Anisolepis* sp., and the other composed of Gekkota.

Grasping (Figs. 1–10): The most parsimonious state for the common ancestor is ambiguous. This character has three independent origins in the tree, in *Tropidurus* sp., *Anolis* sp. and *Gekkota*. In this last case, grasping ability is a synapomorphy of the entire group.

## Discussion

**Figure 11:**
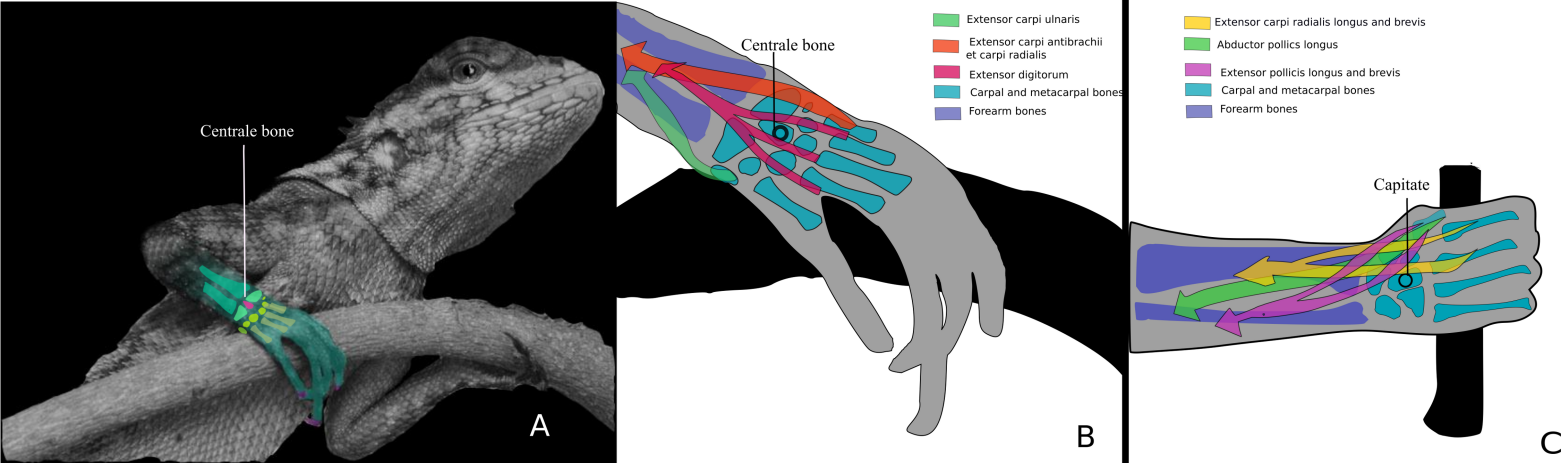
Centrale bone as a keystone in the carpus. (A) *Plica umbra* grasping a narrow branch. On the right hand, the first cleft surrounds the branch. On the left hand, the third cleft surrounds the branch. A detail of the right hand shows the approximate arrangement of the carpal and metacarpal during grasping. Photo: Rafael Balestrin. (B) Detailed diagram of (A). Three muscles are shown: extensor carpi ulnaris (green); extensor ant ebrachii et carpi radialis (orange); extensor digitorum (pink). (C) Human arm showing the relationship between the capitate and the forearm muscles (modified from Neumann (2010)). Three muscles are shown: extensor carpi radialis longus and brevis (yellow); abductor pollicis longus (green); extensor pollicis longus and brevis (pink). Note that the centrale bone in grasping lizards hands exhibit the same form, position and probably mechanical role as the capitate in human hands. Both, the centrale and capitate probably act as keystones and control the axis of rotation.

Our analyses yielded six continuous variables associated with grasping abilities: two belong to the carpal bones, two belong to the metacarpals and two belong to the phalanges. Five of these show adaptive trends once their evolutionary histories have been traced. The history of the width of the centrale bone can be interpreted as a convergent trend towards a narrower bone structure and consequently enhanced grasping capabilities. This bone is of particular importance in determining the articulation between the carpal bones. In the complex grasping hands of humans, the capitate bone (distal carpal 3) occupies a central location within the wrist, articulating with seven surrounding bones. A rigid articulation allows the capitate and the third metacarpal to function as a single column, providing significant longitudinal stability to the entire wrist and hand. The axis of rotation for all wrist motions passes through the capitate (Neumann, 2010). The position and form of the capitate and its related muscles directly correlate with the centrale bone in grasping lizards (Fontanarrosa & Abdala, 2014; this work) (Fig. 11), as both function as keystones for the proximal transverse arch of the hand (Neumann, 2010; Fontanarrosa & Abdala, 2014). Thorington & Darrow (2000) showed that enlargement of the centrale in arboreal sciurids reduces the extent of the articulation between the scapholunate (radiale + intermedium) and the capitate, or between the scapholunate and the lesser multangular (distal carpal 2). Fontanarrosa & Abdala (2014) found that the centrale bone of lizards could have four different locations within the proximal carpalia, proving that, unlike sciurids, the centrale bone in lizards modifies the articulation of the proximal carpals more so than the distal ones. The evolutionary history provides clear evidence for the convergent nature of narrow centrale bones: *Anolis*, an emblematic genus of graspers, exhibits narrow centrale bones, as well as *Tropidurus*, many species of which are arboreal and whose hands are capable of some degree of grasping. Remarkably, *Iguana* and *Physignatus*, two lizard genera that lack grasping abilities, also have narrow centrale bones. This particular character, which partially supports our first prediction, could suggest a stronger link with climbing than grasping abilities. Meanwhile Gekkota tend to have a narrow centrale bone and, although none of their representative taxa considered in this work are specifically arboreal, geckos have a morphology that allows them to occupy many of the arboreal microhabitats, including the narrow branches. Most of the Teiioidea present a wider centrale, a feature that seems to be derived in this part of the cladogram, and independently acquired by *Stenocercus* sp.

Exploitation of narrow branch niches has been pervasively linked to the origin of grasping (Sussman, 1991; Abdala et al., 2009; Fabre et al., 2013; Sustaita et al., 2013) and therefore hand mobility. The presence of a large sesamoid bone in the tendinous flexor plate of the palmar side of the hand may limit movement of this tendon and maintain the hand flat (Manzano, Abdala & Herrel, 2008; Abdala et al., 2009; Sustaita et al., 2013). Our results seem to indicate that taxa without the ability to grasp exhibit either intermediate or high palmar sesamoid length. It should be considered that traits related to grasping, particularly the lack of the palmar sesamoid, also tend to increase the mobility of the manus (Abdala et al., 2009). The absence or reduction of a palmar sesamoid leaves a flexible tendinous sheet on the palm of the hand, which is crucial for animals that need to maintain close substrate contact to maximize the adhesive capacity of digit-pads, such as most geckos, anoles and many scincids (Cartmill, 1985; Autumn et al., 2000; Autumn et al., 2002; Autumn & Peattie, 2002; Abdala et al., 2009). A large rigid plate would likely interfere with the hyperextension necessary to release the digital pads from the substrate (Russell, 1975; Abdala et al., 2009), since the deployment of the adhesive system is activated by the musculoskeletal complex (Russell & Oetelaar, 2015). Our results regarding the palmar sesamoid are not conclusive, as they display a range of intermediate values shared by grasping and non-grasping lizards, therefore preventing us from definitively accepting or rejecting our initial hypothesis; however some inferences can be made if both the centrale width and palmar sesamoid length are considered together. A large palmar sesamoid prevents grasping completely (Abdala et al., 2009; Sustaita et al., 2013; Fontanarrosa & Abdala, 2014) and renders the centrale bone width irrelevant in the context of manual abilities. A reduced palmar sesamoid, however, makes the centrale bone size decisive for grasping abilities. When present and reduced, the palmar sesamoid requires a highly attenuated centrale bone to enable grasping as it must counterbalance the palmar sesamoid (a structure, as previously mentioned, that prevents the palmar flexion). When the palmar sesamoid is absent, the centrale bone can exhibit a wider range of sizes within the low and intermediate values and the hand still retains its grasping abilities.

A widely (30°–40°) divergent angle between the first and the rest of the digits of the hand is considered a diagnostic feature for arboreality (Flower, 1885; Gould, 1977; Endo et al., 2001; Sheil & Alamillo, 2005; Salesa et al., 2006; Manzano, Abdala & Herrel, 2008; Pouydebat et al., 2008; Pouydebat et al., 2009; Pouydebat et al., 2011; Fröbisch & Reisz, 2009). The greatest divergence angles between digit one and five was detected in geckos, specifically the pad-bearing geckos *Thecadactylus* sp. and *Phyllopezus* sp. (Fig. 7, red range). These particular taxa present angles that can reach up to 180° (Russell, 1986). Due to these obtuse angles, manual digits radiate around a broad arc (Russell & Bauer, 1989). The radiating pattern of the gecko’s digits has been associated with the necessity of grasping in a variety of body orientations (Russell, 1986), and Russell & Oetelaar (2015) have also inferred that the existence of a spread out arc permits variant digital orientations according to distinct body postures. The remainder of the geckos analyzed presented comparably smaller angles (between 60° and 90°) that could be attributed to their gymnodactylid condition. The relatively small angles are probably related to the secondary simplification of the adhesive system associated with reversion to a terrestrial lifestyle (Johnson, Russell & Bauer, 2005; Higham et al., 2014). Interestingly, all the considered forest dwelling gymnodatylid geckos inhabit either the lower environments of large trees or deserts with no arboreal cover (Vanzolini, 1968; Köhler & Vesely, 2011; Recoder et al., 2012; Burgos Gallardo, 2013), natural contexts in which prehensility does not have an obvious adaptive advantage. In contrast, the arboreal taxa *Anolis* sp. and *Physignatus* sp. exhibit hands that can spread to angles greater than 90° (Fig. 7, green range). Most anoles lizards explore the narrow branch niches using their grasping hands, while *Physignatus* utilizes its interlocking claws for gripping (Cartmill, 1985; Biewener, 2003; Tulli et al., 2009). Both strategies are enhanced by a greater divergence angle between digits that allows the palm to be spread wider and therefore make better contact with the substrate, enabling safer and more versatile branch locomotion. Our results do not allow us to infer that a greater divergence angle is an adaptation exclusively for grasping hands.

The feet of many prehensile vertebrates have digits that point in distinct directions and are separated by a cleft. Narrow branches can be gripped in the cleft of opposing sets of digits (Cartmill, 1985; Abdala et al., 2014). This concept of foot clefts can likewise be used in describing hands. The large angle of divergence present in the hand of geckos would allow them to have at least four clefts, one in each inter-digital space, rendering their hands highly efficient grasping devices (Figs. 2A, 2B and 10A).

Typically the relative length of skeletal limb elements is associated with differences in locomotive abilities (Lemelin & Schmitt, 1998; Patel, 2010; Kirk et al., 2008; Almécija Samers & Jungers, 2015). Longer metapodials are associated with terrestrial locomotion in cursorial and digitigrade animals (Patel, 2010), while Fröbisch & Reisz (2009) propose that the massive, truncated metapodial I of the Permian synapsid *Suminia* is an indicator of arboreality. Following the same rationale, Martill, Tischlinger & Longrich (2015) suggest that the recently discovered four limbed Cretaceous snake *Tetrapodophis amplectus* would have had grasping abilities based on its short metapodials and hyperelongated penultimate phalanges. Our analyses showed that metacarpal I and the fourth phalanx of digit four tend to be narrow in grasping species, although the lengths of the metapodial bones were not recovered by the best model. Nevertheless, considering that all variables were size corrected using the geometric mean, the width of metacarpal I conveys important information regarding the proportions of the bone: narrow metacarpals and narrow phalanges are also slender ones. Some morphometric features of the grasping lizard hand skeleton are therefore more akin to cursorial mammals (Patel, 2010) than to arboreal taxa, as described by Fröbisch & Reisz (2009).

The character mapping shows that longer phalanges appear independently in grasping taxa, reinforcing the conjecture of their adaptive advantage in arboreal lizards, and supporting our fourth prediction. Elongated phalanges have also been pervasively linked to climbing in many tetrapod taxa (Arnold, 1998; Fröbisch & Reisz, 2009). Two patterns of skeletal specializations in arboreal tetrapods have been described; one for graspers and another for clingers, both of which exhibit elongated phalanges (Fröbisch & Reisz, 2009). Other authors have shown that longer manual proximal phalanges and longer digits in general, relative to metacarpal length, are traits that allow primates to grasp small diameter arboreal substrates (Kirk et al., 2008). Our data show that arboreal lizards tend to exhibit the same hand characteristics described for other arboreal tetrapod taxa. These results allow us to infer that certain morphological traits linked to prehensility were acquired very early in the evolutionary history of tetrapods by means of a common phenotypic adaptive strategy: lengthening of the long bones (Gould, 1977; Wallace, 2010).

*Stenocercus* presents a combination of striking characteristics that give a general impression of stockiness: short fourth phalanx of the fourth digit, short first phalanx of the fifth digit, and large palmar sesamoid; all of which are probably linked to its ground dweller locomotor mode (Suárez Cortés, 2011; Perez Daza & Castillo Morales, 2011). On the contrary, *Physignatus* tends to be of slighter build in spite of being included in the phylogenetic group agamids, which is generally characterized by its stocky form (Honda et al., 2000). In comparison with others in the group, *Physignatus* exhibits a longer fourth phalanx of the fourth digit, the narrowest centrale, a longer first phalanx of the fifth digit, and a narrower first metacarpal than its relatives. These particular characteristics observed in *Physignatus* have also been independently acquired in *Iguana* and are probably related to the climbing abilities present in both taxa.

### Morphological strategies of a grasping hand

Results of our study indicate that grasping in lizards can be performed with hands exhibiting at least two different independently originated combinations of bones. The first is a combination of a highly elongated centrale bone, reduced palmar sesamoid, and slender metacarpal V and phalanges, such as exhibited by *Anolis* sp. and *Tropidurus* sp. The second is composed of an elongated centrale bone, lack of a palmar sesamoid, and slender metacarpal V and phalanges, such as exhibited by geckos. Our data suggest that the morphological distinction between graspers and non-graspers is an artificial one. Even when considering the palmar sesamoid, we detected a morphological continuum within which a new ability is generated.

Our results support the hypothesis of the nested origin of grasping abilities within arboreality, as in other tetrapod clades (Sussman, 1991; Fabre et al., 2013; Sustaita et al., 2013). Thus, the manifestation of grasping abilities as a response to locomotive selective pressure in the context of an narrow-branch eco-space could also enable other biological roles such as grasping-dependent as prey handling.

## Supplemental Information

10.7717/peerj.1978/supp-1 Data S1 Raw Data Morphometric data set.

## Additional Information and Declarations

### Data Availability

The following information was supplied regarding data availability: The raw data has been supplied as Data S1.
